# Duodenal Squamous Cell Carcinoma From Metastatic Spread of Head and Neck Cancer: A Case Report

**DOI:** 10.7759/cureus.69826

**Published:** 2024-09-20

**Authors:** Milaris M Sanchez-Cordero, Felix Rivera Troia, Fernando J Ocasio Villa

**Affiliations:** 1 Internal Medicine, Mayaguez Medical Center, Mayaguez, PRI; 2 Surgery, University of Medicine & Health Science, Bassettiere, KNA; 3 Genetics, Ponce Health Sciences University, Ponce, PRI; 4 Genetics, Mayaguez Medical Center, Mayaguez, PRI; 5 Genetics, Western Oncology Cancer Center, Mayaguez, PRI

**Keywords:** cancer metastasis, duodenal squamous cell carcinoma, head and neck cancer (h&n cancer), oncology, squamous cells carcinoma

## Abstract

We present the case of a 69-year-old man with squamous cell carcinoma (SCC) of the duodenum, which was identified as a metastatic lesion stemming from recurrent head and neck cancer (HNC). The patient, who had a history of hypertension, came to the emergency department with burning abdominal pain that had persisted for a week. He reported being well until he suddenly experienced lower abdominal pain, accompanied by reduced appetite, nausea, and post-meal vomiting. An abdominal CT scan revealed a high-grade mechanical obstruction of the small bowel, with a transition point in the right lower quadrant. A biopsy confirmed that the mass was a moderately differentiated metastatic keratinizing squamous cell carcinoma. Metastasis of head and neck squamous cell carcinoma (HNSCC) to the duodenum is rare, likely due to the unique anatomy of the area, which lacks significant lymphatic drainage. This case brings to our attention the need to consider atypical metastatic sites in patients with HNSCC and highlights the importance of advanced imaging and immunotherapy in the diagnosis and management of such metastases.

## Introduction

Squamous cell carcinoma (SCC) is a prevalent cancer in the head and neck area. While it rarely metastasizes, it can exhibit local aggressiveness and invade adjacent tissues [1]. It is part of a group of malignancies called Head and Neck Squamous Cell Carcinoma (HNSCC). This larger group of cancers usually involves the oral cavity, pharynx, hypopharynx, larynx, nasal cavity, and salivary gland tissues. The estimated incidence of HNSCC is around 890,000 per year and contributes to roughly 4.5% of cancer diagnoses and deaths [2]. Major risk factors have been linked to this disease, including tobacco smoking, alcohol use, and human papillomavirus (HPV). Studies have shown that tobacco use alone in people who have never drunk alcohol is associated with an increased risk of HNC with an odds ratio (OR) of 2.13 and a 95% confidence interval (CI) = 1.53, 2.98 [3]. Furthermore, it seems the concomitant use of tobacco and alcohol is linked to approximately 72% of head and neck cancers (HNC) worldwide [4].

HNSCC is primarily spread via the lymphatic system, with the most common site of metastasis being regional lymph nodes near the area of the lesion. The likelihood of lymph node metastasis can be estimated based on tumor differentiation, the size and depth of invasion, and capillary lymphatics. Additionally, the risk of lymphatic spread is heightened by tumor recurrence. Distant spread, which is defined as the spread of the tumor to other organs, is relatively rare in HNSCC as compared to other tumors and is associated with poorer prognosis. However, it is mostly seen in nasopharyngeal and hypopharyngeal cancers [5].

Metastatic or primary SCC in the gastrointestinal tract is exceedingly rare, with only a few cases documented in the literature. However, there is a rare case of a 74-year-old female patient diagnosed with duodenal squamous cell carcinoma (SCC) as a metastatic lesion from recurrent head and neck cancer (HNC). Based on her symptoms, an esophagogastroduodenoscopy was ordered and revealed an ulcerated mass in the third portion of the duodenum. A subsequent biopsy confirmed that the mass was a metastatic poorly differentiated SCC [6]. Another case documented gastric and duodenal metastases in a patient who presented with dysphagia. Subsequent investigation revealed that these metastases originated from a primary SCC of the lung [7]. Metastasis of HNSCC to the duodenum is exceedingly rare, likely due to the anatomical location and limited lymphatic drainage in this area, which is why we aim to emphasize the importance of considering atypical metastatic sites in patients with HNSCC.

## Case presentation

This is the case of a 69-year-old patient with a past medical history of hypertension who came to the emergency department (ED) with abdominal pain of one week's duration. The patient reported that the pain was intermittent and worsened with eating. Associated symptoms included constipation, nausea, and vomiting. The patient reported decreased appetite due to these symptoms and mentioned experiencing similar symptoms two months ago, which suddenly resolved. The patient was a former smoker for approximately 30 years and quit 10 years ago.

**Figure 1:**
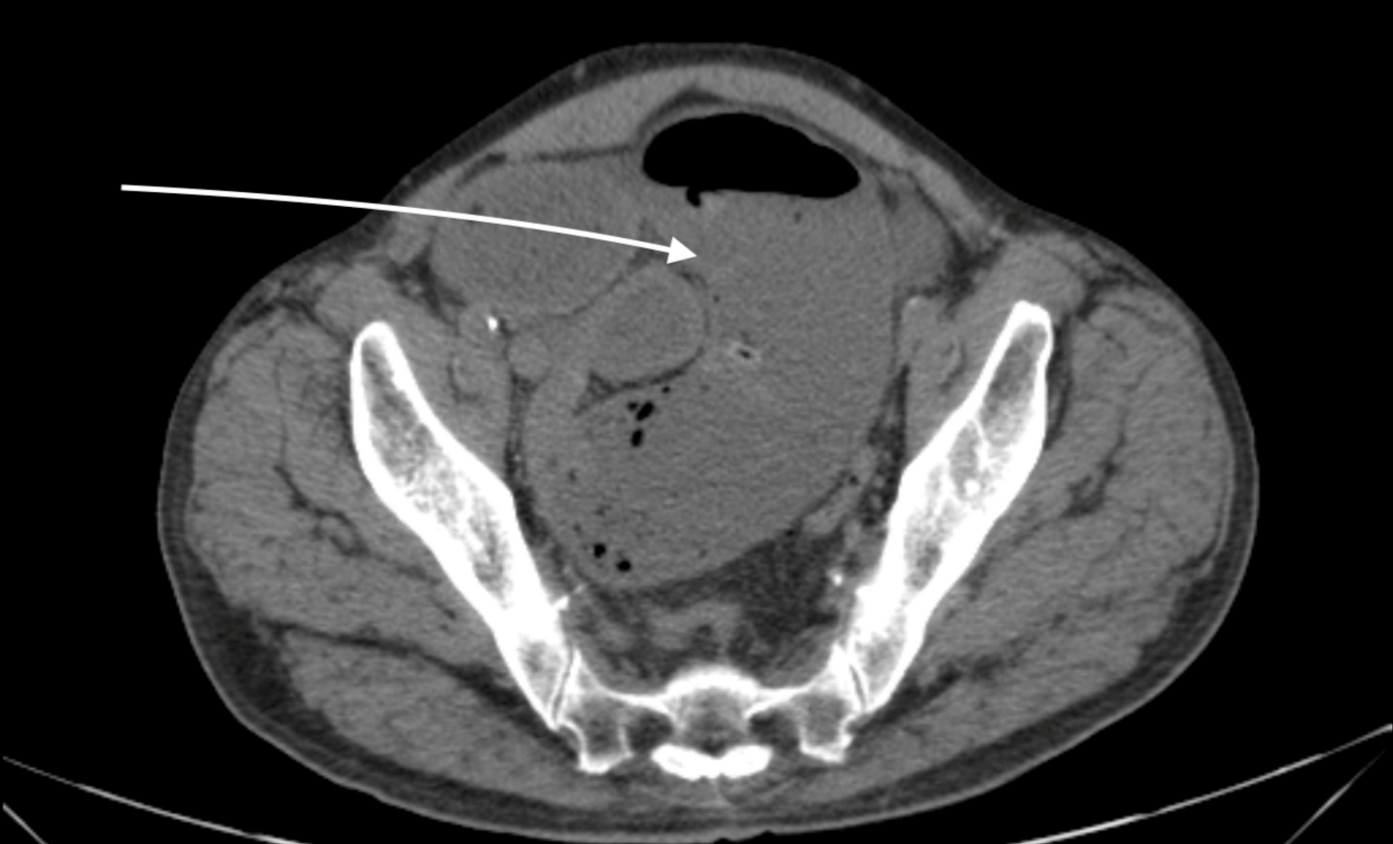
Axial view of abdominal CT scan showing diffuse small bowel distention measuring up to 5.3 cm in the pelvis, with an abrupt change in caliber in the right lower quadrant The terminal ileum collapsed.

Upon examination, the patient was found to have hypoactive bowel sounds, tenderness to palpation, and abdominal distention. An abdominal-pelvic computed tomography (CT) scan showed a high-grade mechanical small bowel obstruction (Figure 1) and surgical services were consulted. The patient underwent a tumor resection in the ileum with end-to-end anastomosis, and the specimen was sent to pathology. The results returned positive for moderately differentiated metastatic keratinizing squamous cell carcinoma. The patient was evaluated by oncology services and started on chemotherapy and immunotherapy. Next-generation sequencing revealed PDL-1 expression, and the patient was continued on pembrolizumab.

**Figure 2:**
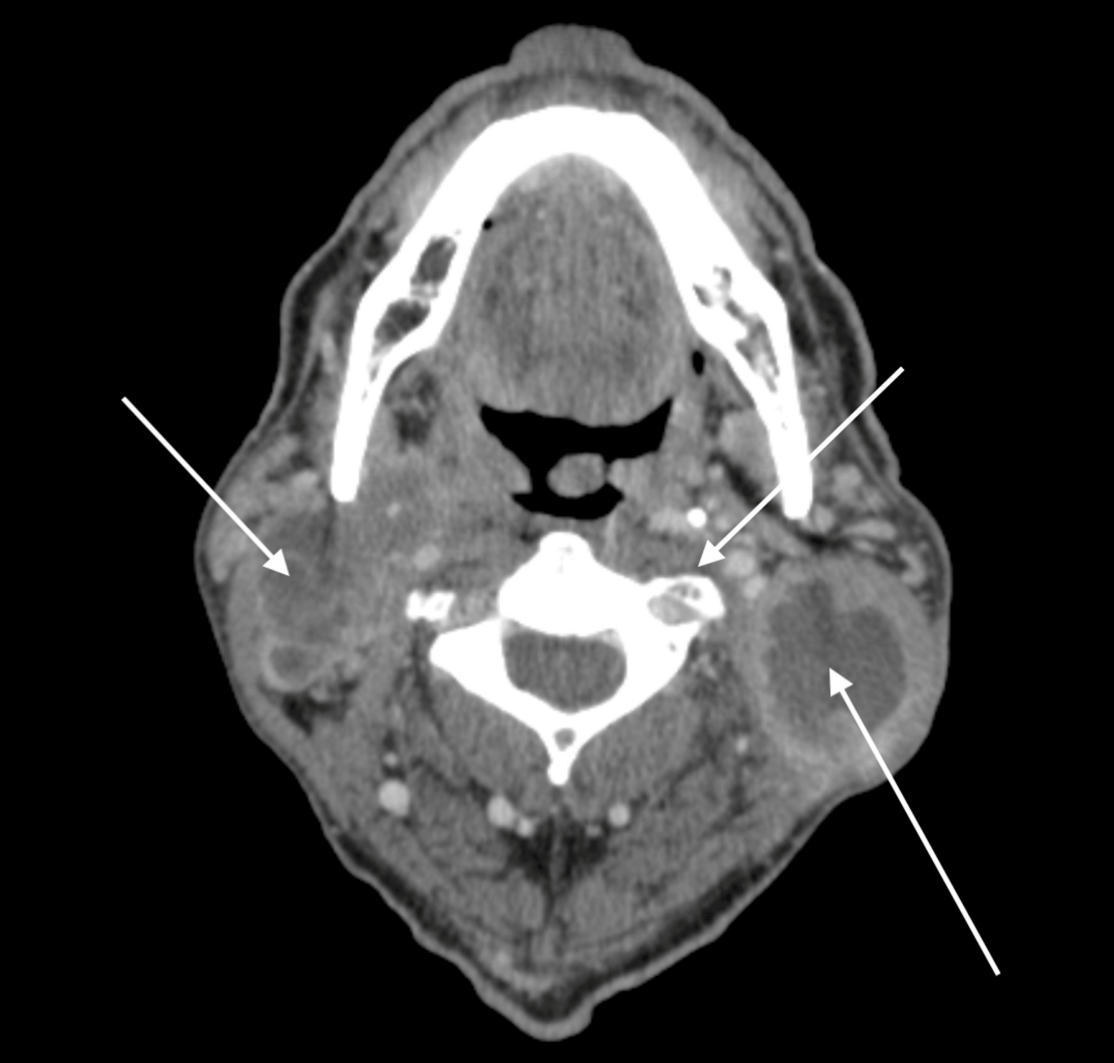
Axial view of head CT scan showing a hypodense laryngeal mass measuring 2.4 x 1.0 x 2.3 cm extending inferiorly into the left aryepiglottic fold and left vocal cord and extensive bilateral necrotic cervical lymphadenopathy measuring up to 5.7 x 3.6 cm

Further investigation determined that the patient had stage IV head and neck cancer on CT imaging (Figure 2).

One month later, the patient presented with back pain, which was found to be due to bone metastasis and worsening lung function, necessitating a thoracentesis. The thoracentesis culture returned negative. The procedure revealed an exudate-type effusion, according to Light's criteria, which correlates with malignant effusion. The patient was treated with a right-sided draining catheter by an interventional radiologist and was discharged home. The patient then returned with shortness of breath and was found to have bacteremia for which he was treated accordingly. Complications included hyponatremia, which nephrology services managed. After stabilization, the patient was discharged to hospice services with supplemental oxygen.

## Discussion

Head and neck squamous cell carcinomas (HNSCCs) are the most prevalent cancers in the head and neck region, arising from the mucosal epithelium of the oral cavity, pharynx, and larynx [2]. They are the sixth most common cancer globally, with 890,000 new cases and 450,000 deaths reported in 2018 [8]. The incidence of HNSCC is rising, projected to increase by 30% by 2030 [9]. HNSCC is commonly linked to tobacco use, excessive alcohol consumption, and HPV infection, particularly with strains HPV-16 and HPV-18. Men are two to four times more likely than women to develop HNSCC, with a median diagnosis age of 66 for non-virally associated cases [2,8]. No effective screening strategy exists, so early detection relies on careful physical examination. Treatment typically involves surgical resection for oral cavity tumors and chemoradiation for pharyngeal or laryngeal cancers.

HNSCC tumors, particularly in advanced stages, show increased PD-L1 expression, which reduces the cytolytic function of T cells [10]. Similarly, myeloid-derived suppressor cells (MDSCs) and T regulatory cells within the HNSCC tumor microenvironment (TME) express PD-L1 and the immunosuppressive molecule cytotoxic T lymphocyte antigen 4 (CTLA-4), respectively [2,10]. In HPV-positive HNSCC, viral proteins E5, E6, and E7 contribute to immune evasion by altering gene and protein expression in tumor cells. The frequent loss of TRAF3, a gene involved in antiviral immunity, in HPV-positive HNSCC may further promote immune evasion [11]. PD-L1 expression levels in the tumor are associated with a higher likelihood of clinical benefit, which led to the 2019 approval of pembrolizumab as first-line therapy for patients with HNSCC whose tumors have a combined positive score (CPS) of ≥1 [12]. Pembrolizumab enhances the immune system’s ability to fight HNSCC by blocking the PD-1/PD-L1 interaction, which can be effective even in rare metastatic sites like the duodenum. However, the unique immune environment of the GI tract, along with the potential for immune-related adverse events (irAEs), such as colitis or enteritis, must be carefully managed during treatment [13].

Small bowel cancer is an uncommon disease, comprising only 2% of all gastrointestinal tract cancers in the United States, with duodenal cancer making up less than 0.5% [14]. Clinical symptoms of small intestine cancer are often atypical, leading to a late-stage diagnosis. Risk factors for developing duodenal cancer include genetic syndromes, such as familial adenomatous polyposis, Lynch syndrome, and Muir-Torre syndrome, and other syndromes like Gardner's, celiac, and Crohn's disease [14]. This case report underscores the rare occurrence of duodenal metastasis in patients with HNSCC, which typically metastasizes to the lungs, bones, and liver [15]. Metastasis to the gastrointestinal tract, particularly the duodenum, is uncommon. While the pathophysiology of duodenal metastasis from HNSCC is not fully understood, several mechanisms have been proposed for how metastases might spread to the small intestine. These include peritoneal dissemination, direct extension from intra-abdominal cancer, and hematogenous or lymphatic spread [6].

In this case, the patient’s smoking history likely contributed to the development of SCC, given that tobacco is a well-established risk factor [16]. Considering the patient's age, weight loss, and persistent vomiting, he might have benefited from low-dose CT scan screening for lung cancer, as well as a colonoscopy and esophagogastroduodenoscopy. Although there are no current screening guidelines that have been proven to improve survival rates in HNSCC, clinicians should remain vigilant with high-risk patients and ensure adherence to guideline-based, age-appropriate cancer screening.

## Conclusions

This case report illustrates the rare and complex nature of metastatic squamous cell carcinoma (SCC) originating from head and neck cancer (HNC) with an unusual presentation in the duodenum. Our patient, a 69-year-old male with a history of recurrent laryngeal SCC, exhibited a high-grade mechanical small bowel obstruction due to metastatic involvement of the duodenum. The diagnosis of duodenal metastasis from HNSCC is infrequent, underscoring the importance of considering atypical metastatic sites in patients with HNSCC. Advanced imaging techniques, such as CT scans, played a crucial role in identifying the mechanical obstruction and guiding subsequent management. The biopsy confirmed the presence of moderately differentiated metastatic keratinizing squamous cell carcinoma, which was subsequently treated with pembrolizumab, reflecting the effectiveness of immunotherapy in managing even rare metastatic sites.

This case emphasizes the need for heightened awareness among clinicians regarding the potential for atypical metastasis in patients with HNSCC, particularly given the rising incidence and complexity of these cancers. Although duodenal metastasis from HNSCC is rare, it highlights the importance of a comprehensive diagnostic approach and the role of cutting-edge therapies in managing such complex cases. Future research should focus on understanding the mechanisms behind atypical metastasis and refining screening strategies to detect metastases early, potentially improving patient outcomes. This case contributes to the growing body of evidence on rare metastatic patterns in HNSCC and underscores the importance of continued vigilance and innovation in the management of these challenging malignancies.
